# Behavioral Trigger Identification in Recurrent Postpartum Chest Pain After Negative Cardiopulmonary Evaluation: A Case Report

**DOI:** 10.5811/cpcem.65753

**Published:** 2026-10-13

**Authors:** Aditya S. Shrivastav, Suhas Tivaskar, Anurag Luharia, Gaurav Mishra

**Affiliations:** * Datta Meghe Institute of Higher Education and Research, Department of Cardiology, Maharashtra, India; † Datta Meghe Institute of Higher Education and Research, Department of Radiology and Imaging Technology, Maharashtra, India; ‡ Datta Meghe Institute of Higher Education and Research, Department of Radiation Therapy, Maharashtra, India; § Datta Meghe Institute of Higher Education and Research, Jawaharlal Nehru Medical College, Department of Radiodiagnosis, Wardha, Maharashtra, India

**Keywords:** chest pain, postpartum period, emergency service, noncardiac chest pain, eating disorders

## Abstract

**Introduction:**

Recurrent chest pain in postpartum patients frequently results in repeated emergency department (ED) visits despite negative cardiopulmonary investigations, creating diagnostic uncertainty and increasing healthcare resource utilization. We report a case highlighting the value of a structured postexclusion assessment to identify clinically relevant behavioral triggers after life-threatening causes have been excluded.

**Case Report:**

A 33-year-old woman, six weeks postpartum, presented repeatedly with nocturnal chest pain, palpitations, and dyspnea. Serial electrocardiograms, cardiac biomarkers, chest imaging, and transthoracic echocardiography were consistently normal, excluding acute cardiopulmonary pathology. Careful reassessment of symptom chronology and behavioral history identified a reproducible temporal association between symptom onset and nocturnal binge-eating episodes. Following outpatient psychiatric and clinical psychological evaluation confirming Diagnostic and Statistical Manual of Mental Disorders, Fifth Edition, diagnostic criteria for binge-eating disorder, targeted behavioral counseling led to a marked reduction in symptom frequency and no further ED presentations during follow-up.

**Conclusion:**

This case demonstrates that, after exclusion of life-threatening cardiopulmonary disease, a structured assessment emphasizing symptom temporality and behavioral triggers can uncover clinically actionable mechanisms underlying recurrent postpartum chest pain. Incorporating this approach into emergency department evaluation may facilitate appropriate management while reducing unnecessary repeat investigations and ED utilization.

## INTRODUCTION

Chest pain accounts for approximately 5% to 8% of all emergency department (ED) visits worldwide and routinely prompts high-intensity investigation because of the need to rapidly exclude life-threatening cardiopulmonary disease.^1^,^2^ Postpartum patients represent a subgroup with particularly high ED utilization, especially during the first weeks following delivery, driven by physiological changes after childbirth, heightened symptom vigilance, and low thresholds for re-presentation when symptoms recur.^3^,^4^ In this population, clinicians must remain alert for serious conditions including pulmonary embolism, peripartum cardiomyopathy, acute coronary syndromes, and other cardiopulmonary complications, which often justify comprehensive diagnostic evaluation.

Although most postpartum chest pain assessments ultimately exclude acute cardiac pathology, repeated presentations are common and contribute substantially to diagnostic workload, resource utilization, and patient distress.^1^,^3^,^5^ For emergency physicians, recurrent chest pain with consistently negative investigations presents a practical challenge: Reassurance alone often fails to prevent return visits, yet clear guidance on next-step assessment once acute pathology has been excluded remains limited.^1^

The absence of structured postexclusion approaches may lead to repeated testing without meaningful progression in clinical understanding or management.^2^,^6^ Such postexclusion assessment typically focuses on symptom temporality, behavioral triggers, and psychosocial context. This case is relevant to emergency clinicians because it illustrates a pattern frequently encountered in practice—recurrent postpartum chest pain, repeated negative cardiopulmonary investigations, and ongoing ED utilization—and demonstrates how a focused post-exclusion clinical assessment can yield clinically actionable insight beyond repeated diagnostic exclusion.^6^ To our knowledge, few published reports have specifically described a structured postexclusion clinical assessment integrating symptom temporality and behavioral triggers in recurrent postpartum chest pain after exclusion of life-threatening cardiopulmonary disease. This case therefore offers a practical diagnostic approach for emergency clinicians managing recurrent postpartum chest pain after negative investigations.

## CASE REPORT

A 33-year-old woman presented repeatedly to the ED over a 6-week period with recurrent chest pain during the early postpartum phase, beginning approximately 6 weeks after an uncomplicated vaginal delivery. Her pregnancy and delivery were uneventful. She had no history of cardiovascular disease, metabolic disorders, or prior similar symptoms.

Chest pain episodes were sudden in onset, predominantly nocturnal, and associated with palpitations and dyspnea. Pain severity was reported as high (up to 8/10 on a numerical rating scale). On several occasions, transient left-sided chest heaviness prompted evaluation for acute coronary syndrome. There was no clear exertional trigger, radiation of pain, or positional or pleuritic component. Despite reassurance following prior evaluations, symptom recurrence frequently prompted repeat ED attendance. Across multiple ED presentations, cardiopulmonary pathology was systematically excluded. Serial electrocardiograms demonstrated normal sinus rhythm without ischemic changes (Image).

*CPC-EM Capsule*

What do we already know about this clinical entity?

*Recurrent postpartum chest pain often leads to repeated emergency department (ED) visits despite negative cardiopulmonary investigations.*

What makes this presentation of disease reportable?

*A structured post-exclusion assessment identified binge-eating disorder as the reproducible behavioral trigger after repeated negative evaluations.*

What is the major learning point?

*Assessment of symptom temporality and behavioral triggers may reveal clinically actionable diagnoses after serious cardiopulmonary disease has been excluded.*

How might this improve emergency medicine practice?

*Structured postexclusion assessment may facilitate appropriate referral and decrease recurrent ED utilization.*

Cardiac biomarkers, including high-sensitivity cardiac troponin I, remained below the institutional diagnostic threshold (reference range, < 26.2 ng/L). Routine laboratory investigations including hemoglobin, white blood cell count, and C-reactive protein were all within normal reference ranges. Transthoracic echocardiography demonstrated normal cardiac structure with preserved left ventricular ejection fraction (approximately 60–65%), normal chamber dimensions, and no regional wall motion abnormalities or pericardial effusion. There was no evidence of postpartum cardiomyopathy or sustained arrhythmia. Pulmonary embolism was considered during evaluation and excluded based on low clinical probability and absence of supportive findings on clinical evaluation and imaging.

During acute assessments, clinicians considered several differential diagnoses including gastroesophageal reflux disease, panic-related symptoms, musculoskeletal chest pain, pulmonary embolism, and cardiomyopathy. These possibilities were evaluated using clinical assessment and appropriate investigations and were considered unlikely based on repeatedly normal findings and the absence of supportive clinical features (Table).

Physical examination was unremarkable, with stable vital signs and a body mass index of 22.4 kg/m^2^. During symptomatic presentations, vital signs remained stable; blood pressure, approximately 118/72 mm Hg; heart rate, approximately 100–110 beats per minute (bpm); respiratory rate, 20 breaths per minute, and SpO_2_, 98% on room air. There were no signs of malnutrition, purging behavior, or restrictive intake.

After repeated negative investigations, we obtained a more detailed history focusing on symptom timing and behavioral patterns. This assessment suggested recurrent nocturnal binge-eating episodes beginning during the postpartum period. The patient was subsequently referred for outpatient psychiatric and clinical psychological evaluation, where a comprehensive behavioral assessment was performed. The diagnosis of binge-eating disorder was established according to Diagnostic and Statistical Manual of Mental Disorders, Fifth Edition (DSM-5), diagnostic criteria by the treating psychiatrist and clinical psychologist. The assessment included a detailed clinical interview focusing on eating patterns, episode frequency, loss of control during binge episodes, associated distress, and symptom chronology. Reconstruction of a symptom–behavior timeline using clinical interview and patient diary review demonstrated that chest pain episodes consistently occurred 30–90 minutes after binge-eating episodes (Figure). Although no validated eating-disorder screening questionnaire was administered, the diagnosis was established through specialist DSM-5–based clinical assessment.

No chest pain was reported on nights without binge eating.

Transient sinus tachycardia (100–110 bpm) was documented during episodes. Episodes were also associated with epigastric fullness, belching, and heightened awareness of cardiac sensations.

Over 6 weeks, repeated ED visits led to multiple negative cardiopulmonary evaluations. Following confirmation of the diagnosis, the patient received behavioral counseling from the multidisciplinary team, including psychoeducation regarding binge-eating disorder, regular meal scheduling, avoidance of nocturnal binge-eating episodes, recognition of symptom triggers, reassurance regarding the absence of underlying cardiopulmonary disease, and continued psychiatric and nutritional follow-up. She was referred for outpatient behavioral and nutritional follow-up to reinforce these strategies.

At 1-month follow-up, binge-eating frequency had decreased substantially (from near-nightly episodes to approximately once weekly), accompanied by a parallel reduction in chest pain episodes (Table). No further ED presentations were reported during the follow-up period.

### Patient Perspective

The patient reported that understanding the relationship between nocturnal binge-eating episodes and chest pain reduced anxiety about a cardiac cause and improved confidence in managing recurrent symptoms.

## DISCUSSION

Recurrent chest pain is a frequent and resource-intensive presentation in the ED, particularly in postpartum patients, requiring exclusion of life-threatening conditions such as pulmonary embolism, peripartum cardiomyopathy, and acute coronary syndromes. Consequently, comprehensive diagnostic evaluation is routinely performed. Despite this, a substantial proportion of patients are ultimately diagnosed with noncardiac chest pain after negative investigations,^6^,^7^ with emerging evidence highlighting the contribution of psychiatric factors in recurrent presentations.^8^ After exclusion of cardiopulmonary pathology, persistent diagnostic uncertainty often remains. In many cases, reassurance alone does not prevent repeat ED visits, resulting in recurrent low-yield investigations, increased healthcare utilization, and ongoing patient distress, a pattern well described in patients with recurrent noncardiac chest pain presenting to ED.^9^ The absence of structured approaches for evaluating symptoms after diagnostic exclusion may contribute to this cycle of repeated testing without meaningful progression in clinical understanding.

The key observation in this case was the consistent temporal association between nocturnal binge-eating episodes and subsequent chest pain. Large-volume postprandial intake may produce chest discomfort through gastric distension, transient gastroesophageal reflux, and autonomic activation, all of which can mimic cardiac chest pain.^6^,^10^ In this case, chest pain reliably occurred within a defined 30- to 90-minute postprandial window and was absent on nights without binge eating, making temporality the most informative clinical observation.

Alternative explanations were also considered. Gastroesophageal reflux disease, esophageal spasm, anxiety-related autonomic symptoms, and panic-related chest pain can all produce similar clinical presentations. However, the reproducible association between binge-eating episodes and symptom onset, combined with improvement following behavioral counseling and reduction in binge episodes, was consistent with a behavioral and postprandial physiological mechanism. In this case, the eating behavior was not formally diagnosed during the ED visit itself but was subsequently evaluated and confirmed during outpatient follow-up after referral. In this case, the diagnosis of binge-eating disorder was established by a psychiatrist and clinical psychologist using DSM-5 diagnostic criteria during outpatient follow-up. Although no validated screening questionnaire was administered, DSM-5–based specialist clinical assessment represents the diagnostic standard for binge-eating disorder in routine clinical practice.

From an emergency medicine perspective, structured postexclusion assessment is essential in recurrent chest pain. A focused evaluation of symptom temporality, nocturnal patterns, recent eating behavior, and associated autonomic symptoms may help identify reproducible symptom–behavior relationships and guide appropriate outpatient referral, reducing repeat low-yield investigations. This case should also be interpreted in light of several limitations. As a single-patient observation, the findings cannot establish a causal relationship between binge-eating behavior and chest pain, and the proposed mechanism remains inferential. Although the diagnosis of binge-eating disorder was established by a psychiatrist and clinical psychologist using DSM-5 diagnostic criteria, no validated eating-disorder screening questionnaire was administered. This may limit direct comparison with studies using standardized screening instruments.

## CONCLUSION

Recurrent postpartum chest pain with negative investigations may reflect reproducible behavioral triggers not identified through routine testing. Structured postexclusion assessment focusing on symptom temporality and behavior can provide actionable clinical insight and reduce repeat ED utilization.

## Figures and Tables

**Figure.**
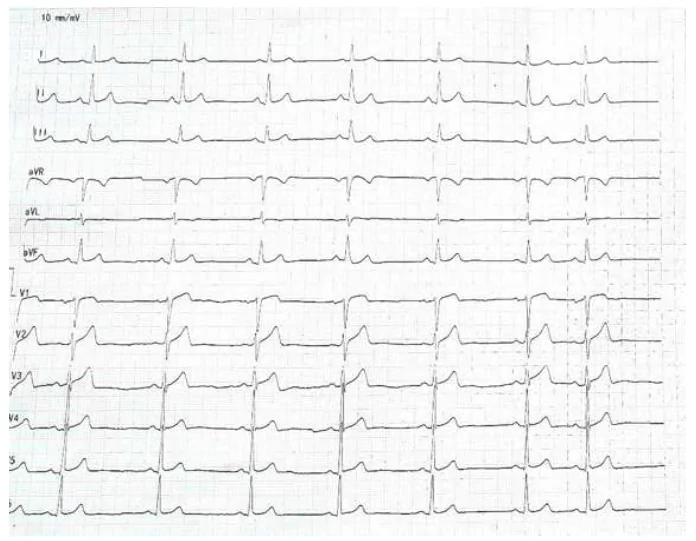
Clinical course timeline across the postpartum period. *ED*, emergency department.

**Image:**
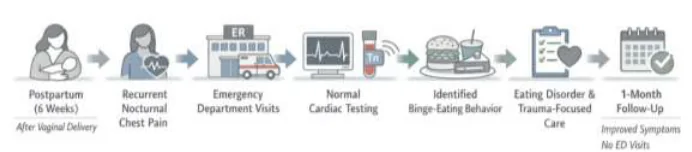
Normal electrocardiogram recorded during chest pain episode. Representative 12-lead electrocardiogram obtained during one of several emergency department presentations.

**Table:** Diagnostic evaluation and clinical course in recurrent postpartum chest pain.

Domain	Key Findings	Assessment/Intervention	Outcome
Acute coronary syndrome	Chest heaviness, palpitations	Serial ECGs, troponin I	Normal ECGs and biomarkers
Postpartum cardiomyopathy	Dyspnea, chest discomfort	Transthoracic echocardiography	Normal ventricular function
Pulmonary embolism	Dyspnea, tachycardia	Clinical probability assessment ± imaging	No supporting findings
Gastroesophageal reflux disease	Postprandial discomfort	Clinical correlation	Not consistent; no response to antacids
Panic-related symptoms	Chest pain, palpitations	Clinical assessment	Temporal pattern favored behavioral etiology
Musculoskeletal pain	Localized tenderness	Physical examination	No reproducible tenderness
Arrhythmia	Palpitations	ECG monitoring	No arrhythmia detected
Behavioral trigger (binge eating)	Nocturnal episodes, postprandial timing	Symptom–behavior correlation, outpatient referral	Reduced binge frequency and chest pain
Follow-up course	Recurrent ED visits initially	Behavioral counseling	No further ED visits

*ECG*, electrocardiogram; *ED*, emergency department.
